# Risk of Urinary Tract Carcinoma among Subjects with Bladder Pain Syndrome/Interstitial Cystitis: A Nationwide Population-Based Study

**DOI:** 10.1155/2018/7495081

**Published:** 2018-06-28

**Authors:** Ming Ping Wu, Hao Lun Luo, Shih Feng Weng, Chung-Han Ho, Michael B. Chancellor, Yao Chi Chuang

**Affiliations:** ^1^Division of Urogynecology and Pelvic Floor Reconstruction, Department of Obstetrics and Gynecology, Chi Mei Medical Center, Tainan, Taiwan; ^2^Center of General Education, Chia-Nan University of Pharmacy and Science, Tainan, Taiwan; ^3^Department of Obstetrics and Gynecology, College of Medicine, Fu-Jen University, Taipei, Taiwan; ^4^Department of Urology, Kaohsiung Chang Gung Memorial Hospital, Chang Gung University College of Medicine, Kaohsiung, Taiwan; ^5^Department of Medical Research, Chi Mei Medical Center, Tainan, Taiwan; ^6^Department of Hospital and Health Care Administration, Chia-Nan University of Pharmacy and Science, Tainan, Taiwan; ^7^Department of Urology, William Beaumont Hospital, Royal Oak, MI, USA

## Abstract

**Objective:**

To investigate the subsequent risks of urinary tract cancers among individuals with bladder pain syndrome/interstitial cystitis (BPS/IC), and gender differences, as well as the effect of associated comorbidity using a population-based administrative database in Taiwan.

**Patients and Methods:**

BPS/IC subjects (10192) and their age- and sex-matched non-BPS/IC control subjects (30576), who had no previous upper urinary tract cancer (UUC), bladder cancer (BC), and prostate cancer (PC), subsequently developed these disorders from the recruited date between 2002 and 2008 and the end of follow-up 2011. A Cox proportional hazards regression model was constructed to estimate the risk of subsequent UUC, BC, and PC following a diagnosis of IC/BPS. The effect of associated comorbidities was measured by Charlson Comorbidity Index (CCI). The risk of outcomes was assessed with Kaplan-Meier curves.

**Results:**

In the BPS/IC subjects, 37 (0.36%) received a diagnosis of BC, and 22 (0.22%) received a diagnosis of UUC; both were significantly higher than the control group, 19 (0.06%) for BC and 30 (0.10%) for UUC. Cox proportional analysis revealed that the adjusted HR for BC and UUC during the follow-up period for patients with IC/BPS was 5.44 (95% CI: 3.10-9.54) and 1.97 (95% CI: 1.13-3.45) than that of comparison subjects. The HRs went up to 5.66 (95% CI: 3.21-9.99) and 2.01 (95% CI: 1.14-3.55) after adjusted by Comorbidity Index (CCI). The male BPS/IC patients have a higher adjusted HR for BC; however, female patients have a higher adjusted HR for both BC and UUC. The adjusted HR for PC has no difference between BPS/IC and control group.

**Conclusion:**

Patients with BPS/IC are at risk of developing BC in both males and females, and UUC in females. This result reminds physicians to evaluate the potential risk of subsequent development of BC and UUC among individuals with BPS/IC.

## 1. Introduction 

Bladder pain syndrome/interstitial cystitis (BPS/IC) is a symptom complex of the bladder characterized by chronic suprapubic/bladder discomfort related to bladder filling, accompanied by urinary frequency, urgency, or nocturia in the absence of infection or another pathological condition [1]. The prevalence of BPS/IC ranged between 0.45% and 12.6% depending on methodological factors used for diagnosis. BPS/IC were more common in women with an estimated female/male ratio ranging from 5:1 to 10:1 [2–4].

Although BPS/IC underlying pathophysiology is incompletely understood, it involves downregulation of tight junctional proteins, increase of urothelial permeability, mast cell activation, and neurogenic inflammation [4]. BPS/IC is a disease of chronic inflammation, which has been linked to the malignant transformation. Keller et al. reported that BPS/IC patients are 2.95 times more likely than comparison subjects to have subsequently received a diagnosis of bladder cancer (BC) [5]. But Keller's study did not include males, nor did they investigate the associations of BPS/IC with other urinary tract cancers and comorbidity effects. In addition, BPS/IC patients have been detected to have a higher level of inflammatory biomarkers in urine and serum [6]. Such inflammatory reaction in urinary tract may share similar etiology in other urinary tract cancers, e.g., upper urinary tract cancer (UUC), which is also a relatively common malignancy in Taiwan. Therefore, we hypothesized that the individuals with BPS/IC may have a higher risk to develop UCC in both genders and prostate cancer (PC) in males; in addition to BC, also, we tried to evaluate the gender difference.

Based on a nationwide population-based database in Taiwan, we conducted this longitudinal cohort study to investigate and explore the subsequent risks of BC, UCC, and prostate cancer (PC) among individuals with bladder pain syndrome/interstitial cystitis (BPS/IC), and gender differences, as well as the effect of associated comorbidity.

## 2. Materials and Methods

### 2.1. Data Source

This study used a data set released by the Taiwan National Health Research Institute (NHRI) in 2011. Taiwan's National Health Insurance (NHI) program was instituted in March 1995 and provides coverage for over 98% of the residents. The NHRI transferred NHI reimbursement data into files for research. The details of National Health Insurance Research Database (NHIRD) were described in our previous study [7]. In addition, hundreds of studies have been published using the NHIRD, so the validity of NHIRD is confirmed [8]. All NHI datasets can be interlinked through each individual personal identification number. To conform to the Personal Information Protection Act, the unique identifiers of the subjects and the institutes have been scrambled cryptographically to assure anonymity. Confidentiality assurances are addressed by abiding by data regulations of the Bureau of NHI, and we have consulted with the Institutional Review Board of Chi Mei Medical Center and obtained a formal written waiver for the need of ethics approval (no. 10202-E07).

## 3. Selection of Patients and Examined Variables

### 3.1. Study Group and Outcomes

We followed the methods of Chuang, YC., Weng, SF., Hsu, YW. et al. “2015” for the enrollment of BPS/IC, whose study confirmed the higher risk of developing mental disorders after the diagnosis of BPS/IC [3]. For cases in this retrospective cohort study, we first identified subjects aged ≥18 years who had received their first-time diagnosis of BPS/IC (ICD-9-CM code 595.1, chronic interstitial cystitis) in an ambulatory care visit (including outpatient departments of hospitals and clinics) from Jan 1st, 2002, to December 31, 2008. To increase the diagnostic validity, the present study only included patients who had at least three diagnoses of BPS/IC coded, whose conditions reflect the clinician's assessment, medication, and outcome evaluation [9]. We excluded those subjects who had ICD-9 codes of BC (188) or UUC (189) or PC (185) before the index entry date. For study cases, we assigned their first date of receiving a diagnosis of BPS/IC as the index date. BPS/IC subjects were identified from the whole population (about 23 millions).

### 3.2. Control Group

For each BPS/IC case, three non-BPS/IC controls were randomly selected from the longitudinal Health Insurance Database 2000 (LHID 2000), a data subset of the National Health Insurance Research Database (NHIRD) that contains all the claim data (from 1996 to 2011) for one million beneficiaries (4.34% of the total population). Non-BPS/IC controls were matched by age, gender, and index date. The index date for the BPS/IC patients was the date of their first registry and the index date for the non-BPS/IC controls was created by matching the date of the BPS/IC subject's index date. Same as BPS/IC group, non-BPS/IC control subjects who were diagnosed with BC, UUC, and PC before index date were excluded.

Each patient was tracked for a 3-year period starting from their index day to identify those who subsequently received a diagnosis as BC, UUC, or PC defined as the events. Demographic data (e.g., gender and age) were recorded. We also recorded comorbid disorders, including hypertension (ICD-9-CM code 401-405), diabetes mellitus (ICD-9-CM code 250), hyperlipidemia (ICD-9-CM code 272), and CKD (ICD-9-CM code 580-588). The criterion to include a comorbid condition was that it should be documented at least once in the inpatient setting or at least three times in the ambulatory setting within one year before the index date. The Charlson Comorbidity Index (CCI) score was also calculated to estimate each individuals' possible health severity level [10].

### 3.3. Measure and Statistical Analysis

The demographic and clinical characteristics were compared between the BPS/IC group and the non-BPS/IC group. Age was classified into three categories: 18–39, 40–60, and >60 years. Pearson's chi-square test was used to compare the difference between BPS/IC group and the non-BPS/IC control group in terms of sociodemographic characteristics and comorbidities. A Cox proportional hazard regression analysis was used to compute the adjusted hazard ratio (HR) for developing outcomes after adjusting for possible confounding factors, such age, gender, DM, HTN, renal, CAD, hyperlipidemia, geographic region, income, and CCI score. The definition of outcome was patients with the incidence of BC, UUC, or PC in the follow-up period from index date to diagnosis date of BC, UUC, or PC. Kaplan-Meier analyses were also used to calculate the cumulative incidence rates of outcomes between two cohorts. The log-rank test was used to analyze the differences in Kaplan-Meier curves. Data are presented as mean (SD), and 95% confidence intervals (CI) are provided, when applicable. Statistical significance was defined as a P value less than 0.05. A two-tailed P value of 0.05 was considered statistically significant. All the analyses were performed with the SAS software version 9.4 (SAS Institute, Cary, NC).

## 4. Results

We followed the methods of Chuang, YC., Weng, SF., Hsu, YW. et al. “2015” for the enrollment of BPS/IC, whose study confirmed the higher risk of developing mental disorders after the diagnosis of BPS/IC [3]. The study included 10192 subjects with BPS/IC and matched 30576 non-BPS/IC subjects. The age and gender distributions were comparable, with a mean age of 49.5 years and 80.8% of women. The prevalence of hypertension, diabetes, CKD, CAD, and hyperlipidemia was significantly higher in BPS/IC than non-BPS/IC groups (Table 1). The finding reflects the reality that IC may associate more comorbidity. The majority of the sampled patients resided in more urbanized areas and in the Northern part of Taiwan. The monthly incomes were of no significant difference between BPS/IC and comparison patients.

During the period, the composite outcome occurred in 37 (0.36%) cases with BC, 22 (0.22%) with UUC, and 11 (0.56%) with PC in the BPS/IC group; on the contrary, there were 19 (0.06%) with BC, 30 (0.10%) with UUC, and 25 (0.43%) with PC in the non-BPS/IC group. The adjusted HR for BC and UUC during the follow-up period for patients with IC/BPS was 5.44 ( 95% CI: 3.10-9.54) and 1.97 (95% CI:1.13-3.45) than that of comparison subjects. The HRs went up to 5.66 (95% CI: 3.21-9.99) and 2.01 (95% CI: 1.14-3.55) after adjusted by Comorbidity Index (CCI). However, the adjusted HR for PC was of no difference between BPS/IC and control group. The male BPS/IC patients have a higher adjusted HR for BC 12.00 (95% CI: 4.31-33.45), than female patients 3.57 (95% CI: 1.77-7.20); however, female patients have a higher adjusted HR for UUC 2.13 (95% CI: 1.11-4.10) than male patients 1.59 (95% CI: 0.50-5.11) (Table 2).

**Figure 1:**
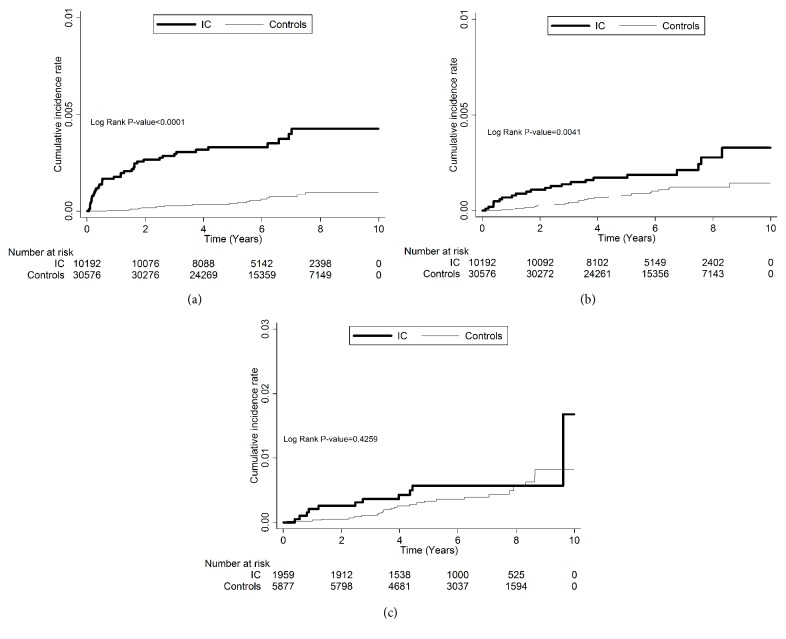
The incidence of bladder cancer (BC), upper urinary tract cancer (UUC), and prostate cancer (PC). (a) BC is significantly increased in patients with interstitial cystitis; (b) UUC is significantly increased in patients with interstitial cystitis; (c) PC is similar in patients with interstitial cystitis or not.

The Kaplan-Meier survival plot (Figure 1) revealed that BPS/IC patients noted have significantly increased bladder cancer and upper urinary tract cancer (p<0.0001 and 0.0041, respectively) compared with control group. This phenomenon cannot be observed between BPS/IC patients and prostate cancer.

## 5. Discussion

Our longitudinal cohort study demonstrated that, after adjusting for age and gender, BPS/IC was associated with increased risks of subsequent development of BC in both males and females and UUC in only females who were initially free of these urological malignancies. Prior researches found the positive association between BC and prior BPS/IC [5]. To the best of our knowledge, this is the first large-scale population-based longitudinal cohort study that investigated the incidence rate of BPS/IC on the subsequent development of UUC and prostate cancer, in addition to BC alone. The current study further revealed the higher risk of developing not only BC in both genders, but also UUC in females after the diagnosis of BPS/IC via a longitudinal cohort study, with implies a potential causal relationship.

BPS/IC is a chronic inflammatory disease. A recent study using bladder biopsies from female subjects with BPS/IC showed upregulation of transcripts involved in the inflammatory and immune cellular signaling systems and downregulation of transcripts responsible for proteins in tight junction pathway in BPS/IC patients with low capacity [9]. Epithelial cells line the interior bladder wall and form a barrier against noxious substances. Once the barrier function is disrupted, it will induce a cascade of inflammatory reaction and symptoms of BPS/IC [11]. The functional relationship between inflammation and cancer is not new. It was hypothesized that some classes of irritants, together with the tissue injury and ensuing inflammation they cause, enhance cell proliferation [12]. Tissue inflammation is an environment rich in inflammatory cells, growth factors, and DNA-damage-promoting agents, all of which might carry neoplastic risk. Therefore, our longitudinal cohort study pointed the need of periodical checkup cystoscopy to rule out the possibility of BC even after the diagnosis of BPS/IC, especially those with hematuria [13]. Urothelial inflammation was obviously in BPS/IC and therefore it can be explained why there was no association between patients with BPS/IC and PC.

The incidence of comorbidities is higher among individuals with BPS/IC, which might indicate a precursor condition, predisposing development of cancer because of systemic inflammation. In Keller et al. study [5], the subjects with BPS/IC had a significantly higher prevalence of 32 medical comorbidities than subjects without BPS/IC. Many of these medical comorbidities are related to systemic inflammation, which might increase the risk of developing BC/UCC. In the hazard ratio evaluation, the risks of BC and UCC went even higher after adjusted by CCI, which implied BPS/IC per se, instead of the health severity, predisposes the subsequent risk of BC and UCC. Therefore, we suggested that patients with BPS/IC should be carefully surveyed for the urinary tract malignancies because of the higher risks found in our national database.

Another important issue is gender effect on the BPS/IC and subsequent risks of urological malignancies. There was no report on the 10-year cumulative incidences of urinary tract cancers in subjects with BPS/IC before, and the male data are always lacking [14, 15]. Therefore, we included both men and women who had no previous urological malignancies. This might reflect the association of BPS/IC on the risk of urinary tract cancers in the general population. The positive correlation between UUC and BPS/IC in females in this national cohort is an interesting finding. Upper urinary tract cancer is a relatively uncommon malignancy but its unusual high prevalence and female predominance is a major public health issue in Taiwan [16]. The most famous carcinogen is aristolochic acid which occupies potentially 60% of UCC with mutational signatures in a single referral center data in Taiwan [17]. Liu M et al. ever reported that aristolochic acid might alter the tight junction of renal epithelial cell line [18]. The mechanism of tight junction deterioration is similar to BPS/IC. To our knowledge, the association between BPS/IC and UUC has never been reported. Surprisingly, we found that BPS/IC was positively associated with female UUC in Taiwan. The causes of development of UUC in BPS/IC patients are not clear to date. This result supports the need of further investigation on aristolochic acid related urothelial inflammation and gender specific cancer susceptibility or higher exposure rate among female. The surveillance for UUC for females diagnosed as BPS/IC might be also necessary for such a high UUC area.

Whether the treatment aimed at BPS/IC may have an effect on the development of BC/UUC remained unanswered in our study; all of the oral and intravesical therapies are not related to cancer development via literature review. Oral treatment including pentosan polysulfate sodium (Elmiron), hydroxyzine hydrochloride, tricyclic antidepressants, and anticholinergics and intravesical therapy including heparin, hyaluronic acid, and botulinum onabotulinumtoxin A (BoNT-A) have been used to relieve the lower urinary tract symptoms in BPS/IC patients in Taiwan. We did not analyze BPS/IC treatment in the current study.

Our study had several limitations. Firstly, the enrollment of the patients with BPS/IC who have at least three times outpatient service claim during the study period may still overdiagnose the BPS/IC [3]. We had no information regarding the accuracy of the codes for BPS/IC in the nationwide population-based study. The diagnosis of BPS/IC is based on specific diagnostic criteria from the cystoscopic or pathological finding with attempt for clinical trial of BPS/IC. Nevertheless, the current enrollment was almost diagnosed by urologists or urogynecologist and might reflect the true situation on the impacts of BPS/IC on the subsequent risk of development of urinary tract carcinoma. Secondly, the misdiagnosis of urothelial carcinoma as BPS/IC at initial presentation is difficult to exclude. Tissot et al. reported that 1% of patients who were considered to have BPS/IC actually had urothelial carcinoma as the cause of symptoms [19]. Utz and Zincke reported on the long-term follow-up of 486 patients treated for IC and found that 1.3% of 224 women and 23% of 53 men subsequently were diagnosed with carcinoma in situ. Both Utz's study and our current finding also confirmed a higher risk of BC for male BPS/IC patients. Furthermore, the current study revealed that BPS/IC has significant association with UUC in females, which would not be related to symptoms of BPS/IC [20]. Secondly, although we have controlled some variables, e.g., age and gender, there are still some other possible confounding factors not yet controlled, such as dietary, obesity, smoking, alcohol, daily activity, and medications, which may link to the development of BC and UUC [21, 22]. Further study with more comorbidities and generating a comorbidity score and stratification might provide a better understanding of BPS/IC with BC/UCC. Nevertheless, the incurable characters of BPS/IC would make the patients stuck to physicians. We suggested that the physicians evaluate the potential risks of subsequent development of BC and UUC among individuals with BPS/IC in the long-term follow-up.

## 6. Conclusion

Patients with BPS/IC are at risk to develop BC in both genders and UUC in females, especially in area with high prevalence of aristolochic acid related disease. These findings provide useful information in malignancy prevention and screening for patients with BPS/IC in clinical practice.

## Figures and Tables

**Table 1:** Patient demography.

	IC	Non-IC	
	n =10192	n =30576	
Characteristic	N (%)	N (%)	p-value
Gender			
Female	8233(80.78)	24699(80.78)	1.0000
Male	1959(19.22)	5877(19.22)	
Average age, mean±SD	49.50±16.73	49.49±16.72	0.9716
Age (years)			
<40	3132(30.73)	9382(30.68)	0.9936
40-60	4399(43.16)	13216(43.22)	
>60	2661(26.11)	7978(26.09)	
DM	842(8.26)	1942(6.35)	<0.0001
CAD	702(6.89)	1139(3.73)	<0.0001
Renal disease	260(2.55)	337(1.10)	<0.0001
Hyperlipidemia	722(7.08)	1463(4.78)	<0.0001
HTN	2026(19.88)	4338(14.19)	<0.0001
CCI score, mean±SD	0.74±1.64	0.38±0.97	<0.0001
Geographic region			
Northern	5699(55.92)	15608(51.05)	<0.0001
Central	1678(16.46)	5428(17.75)	
Southern	2498(24.51)	8857(28.97)	
Eastern	317(3.11)	683(2.23)	
Income, NTD per month			
<$15,840	4260(41.80)	12532(40.99)	0.1525
$15,840~$25,000	3404(33.40)	10180(33.29)	
>$25,000	2528(24.80)	7864(25.72)	

IC: interstitial cystitis, DM: diabetes mellitus, CAD: coronary artery disease, HTN: hypertension; CCI: Charlson Comorbidity Index; NTD: New Taiwan dollar.

**Table 2:** Crude and adjusted hazard ratio for Urotract Cancer in the follow-up period.

	All		Female		Male	
	IC	Non-IC	IC	Non-IC	IC	Non-IC
	N =10192	N =30576	N =8233	N =24699	N =1959	N =5877
Bladder Cancer (n)	37(0.36)	19(0.06)	20(0.24)	14(0.06)	17(0.87)	5(0.09)
Median follow-up time, year	6.04	6.02	6.03	5.99	6.10	6.17
Crude HR (95%CI)	5.84(3.36-10.15)	1.00	4.27(2.16-8.45)	1.00	10.30(3.80-27.93)	1.00
P-value	<0.0001		<.0001		<0.0001	
Adjusted HR1 (95%CI)	5.44(3.10-9.54)	1.00	3.52(1.75-7.08)	1.00	10.57(3.87-28.87)	1.00
P-value	<0.0001		0.0004		<0.0001	
Adjusted HR2 (95%CI)	**5.66(3.21-9.99)**	1.00	**3.57(1.77-7.20)**	1.00	**12.00(4.31-33.45)**	1.00
P-value	<0.0001		0.0004		<0.0001	

Upper urinary tract cancer (n)	22(0.22)	30(0.10)	17(0.21)	22(0.09)	5(0.26)	8(0.14)
Median follow-up time, year	6.05	6.02	6.03	5.99	6.12	6.17
Crude HR (95%CI)	2.19(1.27-3.80)	1.00	2.31(1.22-4.34)	1.00	1.89(0.62-5.78)	1.00
P-value	0.0051		0.0097		0.2640	
Adjusted HR1 (95%CI)	1.97(1.13-3.45)	1.00	2.16(1.13-4.13)	1.00	1.46(0.47-4.54)	1.00
P-value	0.0174		0.0199		0.5143	
Adjusted HR2 (95%CI)	**2.01(1.14-3.55)**	1.00	**2.13(1.11-4.10)**	1.00	**1.59(0.50-5.11)**	1.00
P-value	**0.0158**		**0.0239**		**0.4331**	

Prostate Cancer (n)					11(0.56)	25(0.43)
Median follow-up time, year					6.11	6.16
Crude HR (95%CI)					1.33(0.66-2.71)	1.00
P-value					0.4274	
Adjusted HR1 (95%CI)					1.42(0.69-2.90)	1.00
P-value					0.3400	
Adjusted HR2 (95%CI)					**1.56(0.74-3.30)**	1.00
P-value					**0.2434**	

This table is adjusted by age, gender, DM, HTN, renal disease, CAD, hyperlipidemia, geographic region, and income; CCI score was added in adjusted HR2.
